# Immunoporosis: Immunology of Osteoporosis—Role of T Cells

**DOI:** 10.3389/fimmu.2018.00657

**Published:** 2018-04-05

**Authors:** Rupesh K. Srivastava, Hamid Y. Dar, Pradyumna K. Mishra

**Affiliations:** ^1^Department of Zoology, School of Biological Sciences, Dr. Hari Singh Gour University, Sagar, India; ^2^Department of Biotechnology, All India Institute of Medical Sciences (AIIMS), New Delhi, India; ^3^Department of Molecular Biology, ICMR-National Institute for Research in Environmental Health, Bhopal, India

**Keywords:** immunoporosis, osteoimmunology, bone loss, T lymphocytes, osteoporosis

## Abstract

The role of immune system in various bone pathologies, such as osteoporosis, osteoarthritis, and rheumatoid arthritis is now well established. This had led to the emergence of a modern field of systems biology called as osteoimmunology, an integrated research between fields of immunology and bone biology under one umbrella. Osteoporosis is one of the most common inflammatory bone loss condition with more than 200 million individuals affected worldwide. T helper (Th) cells along with various other immune cells are major players involved in bone homeostasis. In the present review, we specifically discuss the role of various defined T lymphocyte subsets (Th cells comprising Th1, Th2, Th9, Th17, Th22, regulatory T cells, follicular helper T cells, natural killer T cells, γδ T cells, and CD8^+^ T cells) in the pathophysiology of osteoporosis. The study of the specific role of immune system in osteoporosis has now been proposed by our group as “immunoporosis: the immunology of osteoporosis” with special emphasis on the role of various subsets of T lymphocytes. The establishment of this new field had been need of the hour due to the emergence of novel roles of various T cell lymphocytes in accelerated bone loss observed during osteoporosis. Activated T cells either directly or indirectly through the secretion of various cytokines and factors modulate bone health and thereby regulate bone remodeling. Several studies have summarized the role of inflammation in pathogenesis of osteoporosis but very few reports had delineated the precise role of various T cell subsets in the pathobiology of osteoporosis. The present review thus for the first time clearly highlights and summarizes the role of various T lymphocytes in the development and pathophysiology of osteoporosis, giving birth to a new field of biology termed as “immunoporosis”. This novel field will thus provide an overview of the nexus between the cellular components of both bone and immune systems, responsible for the observed bone loss in osteoporosis. A molecular insight into the upcoming and novel field of immunoporosis would thus leads to development of innovative approaches for the prevention and treatment of osteoporosis.

## Introduction

Osteoporosis leads to enhanced rate of fractures and fragility of bones observed in both men and women. It has been estimated that more than 50% of women and 30% of men over the age of 50 years are susceptible for such fractures and bone loss (1). One-third of females and one-fourth of males will be suffering from osteoporosis leading to significant rise in mortality (20–30% associated with first hip fracture) and morbidity (2). Osteoporosis accounts for more than nine million of fractures annually (2). According to the latest International Osteoporosis Foundation report (3), it is estimated that by 2040 the number of osteoporotic patients above age of 50 years will double worldwide from that of 2010 figures of 158 million (3, 4). It has been estimated that by the end of 2025 the economic burden of osteoporosis will reach $25.3 billion in the USA alone (5, 6).

Due to their common developmental niche, both the bone and immune systems work as a close knit functional unit (osteoimmune system), thereby leading to permanent interactions at various anatomical and vascular sites (7). This interaction of immune and skeletal system has now made it clear to the scientific fraternity that there do exist a nexus between these duo-systems. This intricate relationship between bone and immune systems has been fascinating scientists since the early 1970s, paving path for birth of a dedicated field of modern biology called as “osteoimmunology” (8). Dysregulation of immune system has already been related with initiation of different inflammatory autoimmune diseases leading to adverse effects on bone integrity (9). This affects the bone either in a localized way as in case of rheumatoid arthritis (RA) or *via* modulating bone metabolism which regulates key bone cell activities including differentiation. In other cases, immune cells induce changes in key factors or functional components of bone mass regulators, thereby affecting bone health. However, still the interaction between bone and immune system which is not unidirectional is largely unexplored. Indeed, during the recent past it has been observed in various studies that T lymphocytes play an important role in the process of bone remodeling (10).

Bone remodeling is a dynamic equilibrium occurring as a result of interaction between bone cells and bone marrow (BM) cells. Therefore, the lymphocytes residing within the BM form an important component for such process to occur. T cells which account for ~5% of total BM cells are found efficiently in both stromal and parenchymal parts of BM (11). T cells are represented by both CD4^+^ T and CD8^+^ T cell populations. CD4^+^ T cells have a vital role in the function and maintenance of the immune system by helping B cells to enhance production of antibodies along with orchestrating CD8^+^ T cells and other immune cell functions (12). Naive CD4^+^ T cells differentiate into Th1, Th2, Th9, Th17, Th22, regulatory T (Treg) and follicular helper T (T_FH_) depending upon their respective environmental stimuli (13–16). Th17 cells are primarily responsible for initiating and stimulating bone resorption (osteoclastogenesis) (17, 18), while Treg cells are peculiarly associated with inhibition of bone resorption (18–21). Strikingly, not all T cells are osteoclastogenic, as CD8^+^ T cells have recently been reported with bone protecting functions, thereby inhibiting bone loss. CD8^+^ T cells inhibit the process of osteoclastogenesis *via* secretion of various soluble factors, such as osteoprotegerin (OPG) (18) and interferon (IFN)-γ for regulating bone mass (22). Also, several studies have postulated that T cells may simultaneously function as an activator of bone formation (osteoblastogenesis), as they are associated with activation of Wnt signaling pathway in osteoblastic cells (18). In the present review, we will specially focus on the role of various subsets of T lymphocytes, their plasticity, and related unraveled opportunities for future clinical implications in various bone pathologies, with special emphasis on osteoporosis, i.e., “immunoporosis”.

## Bone Cells

Bone, a dynamic organ undergoes continuous remodeling throughout the life of an organism. This task of bone remodeling is meticulously achieved *via* the coordinated synergism between three different types of bone cells, *viz*. osteoclasts (bone eating cells), osteoblasts (bone forming cells) and osteocytes (bone deposition and resorption cells). Osteoblasts originate from mesenchymal stem cells (MSCs) in the BM which also gives rise to chondrocytes, myocytes and adipocytes. Runt-related transcription factor 2 and its target gene the Sp7 transcription factor (known as osterix) are primarily responsible for differentiation of MSCs into osteoblasts (23). In addition, Wnt signaling also plays an important role in the differentiation of osteoblasts (24). Osteocytes are produced as a result of matrix calcification of osteoblasts under the influence of enzyme alkaline phosphatase. Any mechanical strain on the bones is progressed by osteocytes by sending signals through cellular processes (canaliculi’s) to interconnecting osteocytes and also to osteoblasts on surface of bones (25). Osteoclasts are multi-nuclear bone cells which are primarily responsible for bone resorption. They get differentiated from monocytic cell lineages like dendritic cells, macrophages, granulocytes, and microglia. Macrophage colony-stimulating factor (MCSF) is an essential factor required for proliferation and survival of osteoclasts, along with RANK ligand (RANKL) acting *via* its coupling molecule TNF receptor-associated factor 6 (TRAF-6) which leads to their final induction and differentiation (23). For maintaining bone integrity, a dynamic equilibrium is essential between bone forming osteoblasts and bone resorbing osteoclasts. Osteal macrophages (Osteomacs) on the other hand represent a special population of macrophage residing in bony tissues. The term “Osteomacs” was given by Australian researcher Allison Pettit. These are stellate shaped cells and are approximately one-sixth of the cells found in BM, giving rise to a complex networking system (26). They usually get originated from CD68^+^ cell types of macrophage origin (27). Osteomacs are responsible for full functional differentiation and mineralization of osteoblasts during *in vitro* cultures and forms canopy at the site of bone remodeling during *in vivo* conditions. It has been observed that any reduction/alteration of macrophages leads to total loss of endosteal osteomacs and respective osteoblasts, thereby concluding that osteomacs have an important role in maintenance of osteoblast maturity (12, 26, 28–30).

## Bone Remodeling

**Figure 1:**
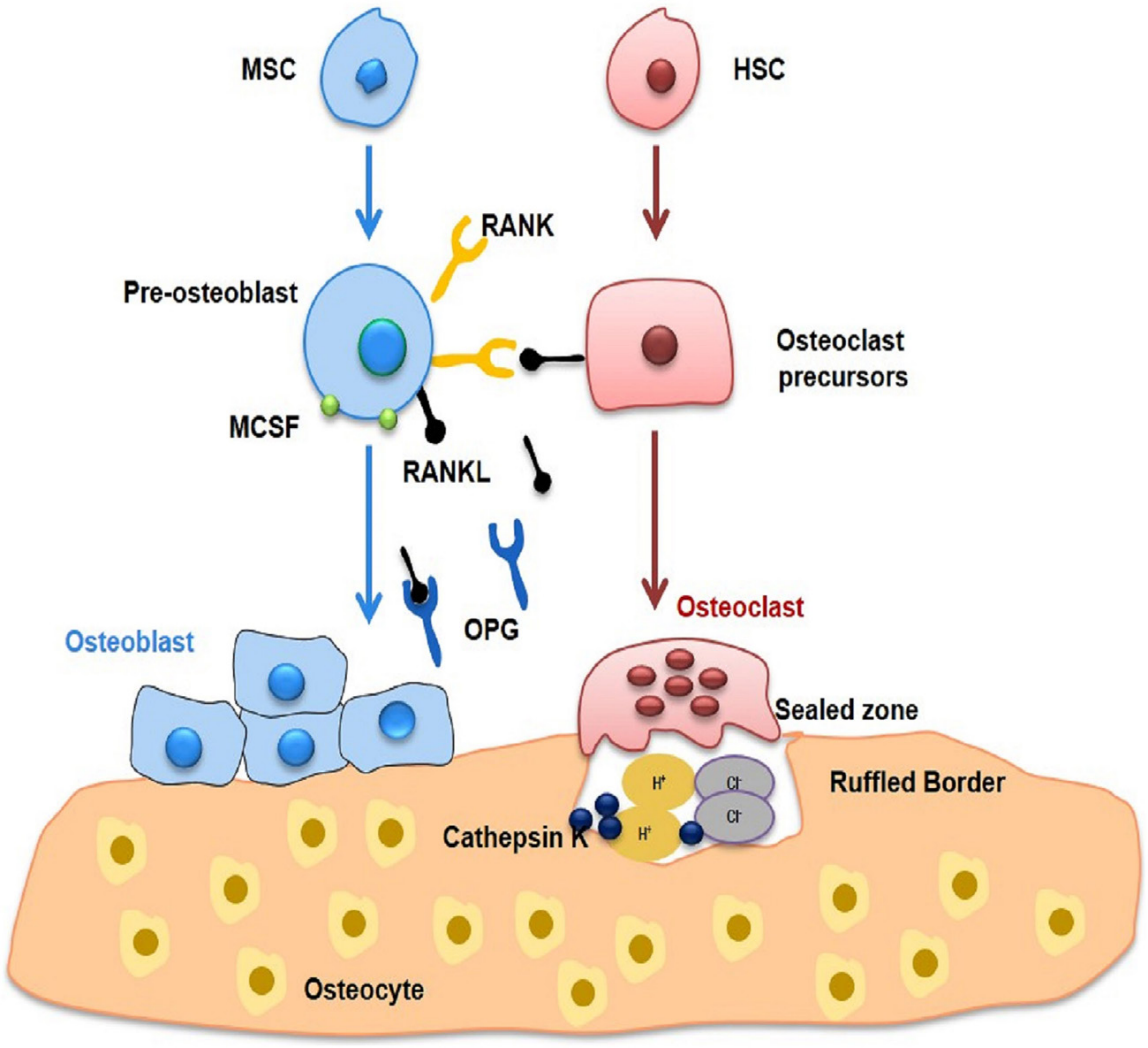
Bone remodelling. Bone undergoes continuous remodelling as a result of interaction between bone-forming osteoblasts and bone-resorbing osteoclasts. Both osteoclasts and osteoblasts lead to fine tuning of osteocytes, which in turn modulates remodelling. Osteoblasts are derivatives of MSCs and produce extracellular bone matrix of type I collagen and non-collagenous proteins, including osteocalcin, osteonectin, and osteopontin. Multinucleated osteoclasts are produced as a result of differentiation of macrophages and monocytes. RANKL in the presence of MCSF is primarily responsible for functioning and activation of osteoclasts leading to bone loss. On the other hand, osteoblasts produce OPG, which inhibits bone loss by inhibiting osteoclastogenesis. MSC, Mesenchymal stem cell; HSC, Hematopoietic stem cell; RANKL, Receptor activator for nuclear factor kappa; OPG, Osteoprotegerin.

Bone remodeling is a dynamic equilibrium resultant of various physiological/mechanical stress accompanied with differential functional adaptations of the bone (31). This dynamicity results in multi-mode interactions between bone resorbing osteoclasts and bone forming osteoblasts (32, 33). Remodeling leads to restoration of bone micro-damages and bone integrity *via* the balancing act in the release of calcium and phosphorus in host. In fact, remodeling corroborates important relationship between bone formation (osteoblastogenesis) and bone resorption (osteoclastogenesis) (Figure 1), regulated at various phases due to impact of immune system on bone cells, neuro-endocrine relationship with bone or a direct interface of osteoclasts and osteoblasts. Bone remodeling comprises of following four-step, *viz*. activation, resorption, reversal and formation (34). Remodeling signals that arise from direct or indirect signals, due to action of hormones [estrogen and parathyroid hormone (PTH)] or structural damage leads to initiation of activation phase. This step is marked by higher apoptotic rate of osteocytes and increased osteoclastogenesis which enhances bone damage (35). The decreasing levels of transforming growth factor beta (TGF-β) resulting from osteoclast apoptosis enhances osteoclastogenesis many folds (36). During activation phase, osteoclast precursor activation occurs as a result of increased activity of protein kinase (C/A) and calcium signaling which is mainly responsible for bone resorption (37). This phase is followed by resorption phase, where osteoclast precursors are recruited by osteoblasts at the site of bone remodeling due to various endocrine signaling or by osteocytes. During this phase, osteoblasts overexpress monocyte chemoattractant protein-1 leading to upregulation of RANKL-induced osteoclastogenesis (38). RANKL is mainly responsible for differentiation and proliferation of osteoclast precursors into multinucleated osteoclasts, thereby enhancing life of mature osteoclasts (38). This results in emergence of an isolated environment called as “sealed zone” due to continuous adhesion of osteoclasts to integrin-binding sites (α_v_β_3_ integrin molecules) on the bone surface (39). The overall result is formation of Howship’s resorption lacunae with increased hydrogen ion concentration (acidic environment) facilitating dissolution of mineralized and organic components of bone (40). The accumulation of cathepsin K enzyme further enhances this rate of bone resorption (41).

Macrophages enhance the expression of osteopontin that leads to deposition of collagen matrix within Howship’s lacunae (42). Next, the bone surface is prepared for bone formation by osteoblasts and osteomacs, primarily responsible for removing different remnants of collagen. The different mechanisms responsible for this coupling phenomenon and for propagating this transition of bone formation to bone resorption has always been a subject of controversy, as earlier studies reported that bone functions as a store house for these coupling molecules and release them accordingly during various steps of bone resorption. During reversal phase, osteoblast precursors differentiate and secrete various molecules that are responsible for development of new bone surfaces. In this phase, various factors like IGF (I and II) and TGF-β are recruited by MSCs to the bone resorption sites (43). Finally, to attain final shape of newly formed bone, hydroxyapatite gets integrated at these newly developed osteoid (43). The bone remodeling phase comes to termination phase when equilibrium is attained between bone formation and bone resorption *via* signals initiated by osteocytes. The loss of sclerostin expression at the end of remodeling cycle instigates the osteoclastogenesis. The mature osteoblasts revert back to bone lining phenotype or undergo apoptosis and subsequently get differentiated into osteocytes. To maintain skeletal structural integrity, bones (both cortical and trabecular) need constant remodeling including repairing various micro-cracks developed due to normal wear and tear. Thus, the important aspects of bone remodeling are the repair, development and maintenance of bone along with functioning as a calcium store house of the host.

## T Lymphocytes and Osteoporosis (Immunoporosis)

**Figure 2:**
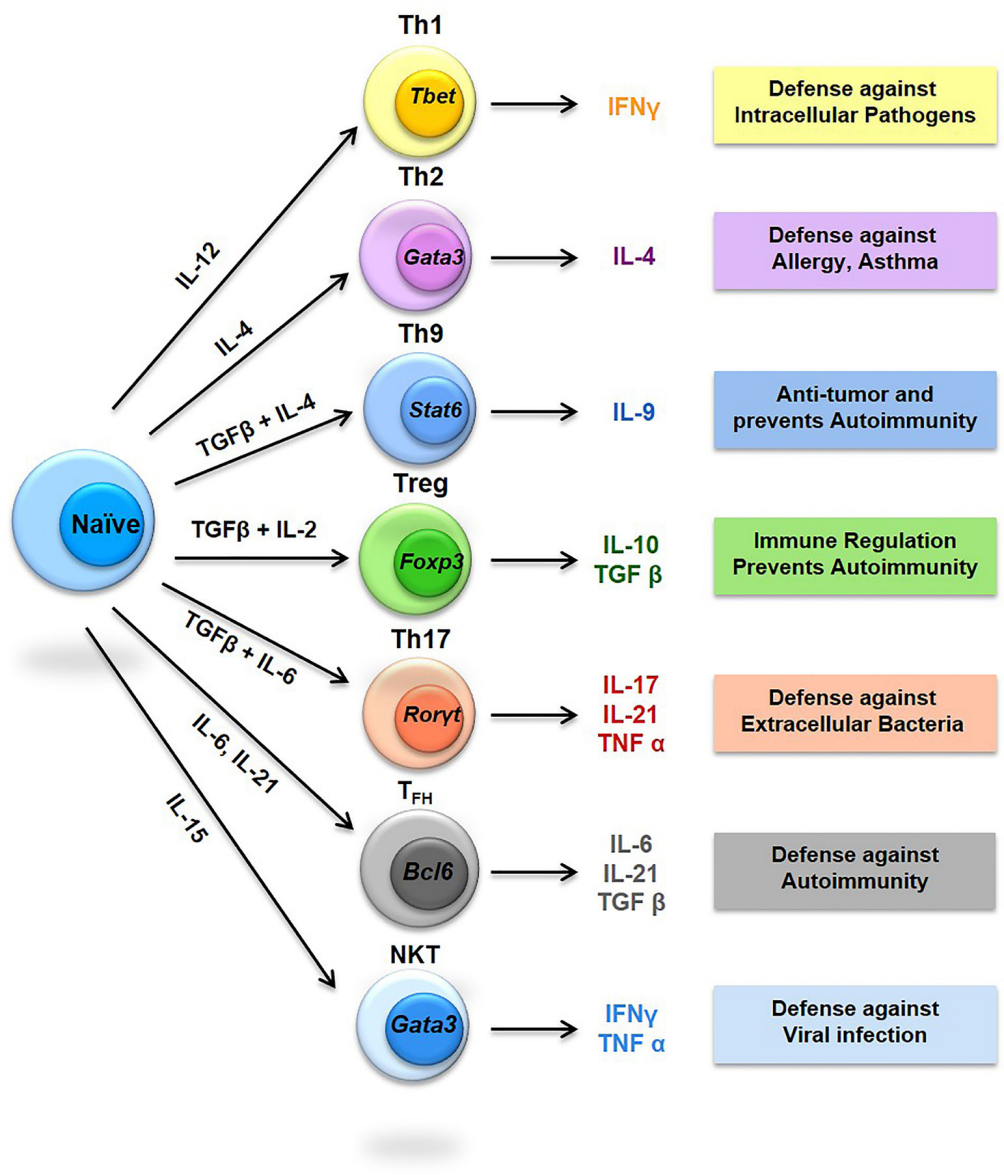
Diversity of T helper (Th) cells. The signature cytokines produced by respective Th cells are shown along with their immunomodulatory properties in boxes. STAT, Signal transducer and activator of transcription; RORγ, RAR-related orphan receptor gamma; Foxp3, Forkhead box P3; BCL6, B-cell lymphoma 6.

Activated T lymphocytes are primary sources of RANKL (44) and TNF-α (45) responsible for bone destruction observed during various pathological (45) and inflammatory conditions (2, 44) (Figure 2). Interestingly, T cells also possess anti-osteoclastogenic properties, as depletion of both CD4^+^ T and CD8^+^ T lymphocytes leads to decreased production of OPG (46–48). Different studies have revealed that T cell deficient nude mice have normal or elevated bone mineral density (45). These studies suggest that T cells have an important role in bone remodeling but the exact link between T cells and osteoclastogenesis particularly in osteoporosis is still not fully elucidated and needs more research thereby giving strong impetus for the emergence of a dedicated novel field to specifically study the role of immune system in osteoporosis, i.e., immunoporosis (with focused emphasis on the role of T lymphocytes).

### Th1 Cells and Osteoporosis

The term Th1 was first given by Tada in 1978 but the clear demonstration of the existence of Th1 cell was provided by Mosmann in 1986 (49, 50). Naive CD4^+^ T cells differentiate into Th1 cells when stimulated by interleukin (IL)-12 resulting in the production of IFN-γ, IL-2, lymphotoxins, TNF-α and granulocyte-macrophage colony-stimulating factor (38, 51). The production of high levels of IFN-γ by Th1 cells induces activation of both phagocytic activity and complement proteins thereby playing an important role in protection against various intracellular pathogens (52). In addition to protection from invading pathogens, Th1 lymphocytes have been associated with development of organ-specific autoimmune diseases (53). Initially, many inflammatory conditions, such as experimental autoimmune encephalomyelitis (EAE), inflammatory bowel disorders (IBD), autoimmune arthritis and collagen-induce arthritis were linked to unchecked Th1 responses (52, 54). Interestingly, Th1 cells do not possess such characteristics thereby making it clear that the osteoclastogenic Th cells might belong to a yet unknown subset (17, 54). Lately, it has been confirmed that yet another recently defined subset of helper T cells (*viz*. Th17 cells) are predominately responsible for causing these inflammation related bone pathologies (55, 56). This new subset thus became the fore runner for the modulation and regulation of bone health. Also, majority of cytokines produced from Th1 cells inhibit osteoclastogenesis (57), even small quantities of IFN-γ can inhibit osteoclastogenesis through degradation of TRAF6 molecule (9) thereby inhibiting bone loss. Together, these compelling findings establish the osteoprotective role of Th1 responses in the pathogenesis of osteoporosis.

### Th2 Cells and Osteoporosis

Like Th1, the term Th2 was also given by Tada in 1978, and later, Mosmann in 1986 (49, 50) provided a full description about Th2 cells. Th2 cells play an important part in host defense against multi-cellular parasites and in protection against allergies and atopic illnesses. Th2 cells are produced by stimulating naive CD4^+^ T cells in the presence of IL-4, leading to production of IL-4, IL-5, IL-10 and IL-13 cytokines (12, 34, 51, 57). Th2 cells were initially believed to be responsible for anti-inflammatory activity in various Th1 cell mediated or Th1 model diseases (57). It was also observed that in case of severity of experimental autoimmune myocarditis inhibition of IL-4 with anti-IL-4 monoclonal antibody reduced the severity of disease (57). The Th2 signature cytokines IL-4, IL-5, and IL-13 have been reported to be associated with inhibition of osteoclastogenesis (58). It has been observed that Th2 cell activation enhances production of PTH, resulting in maintenance of anabolic activity of osteoblasts under various inflammatory conditions (59). Simultaneously, it has also been reported that mice lacking T lymphocytes are unprotected by catabolic activity of PTH (59). Interestingly, Th2 lymphocytes have also been reported to inhibit bone loss by significantly lowering the RANKL/OPG ratio (59). IL-4 has been observed to inhibit bone resorption under both *in vivo* and *in vitro* conditions (60, 61). Low concentrations of Th2 cytokines such as IL-4 and IL-10 have been observed in both the synovial fluid and the peripheral blood of osteoarthritis (OA) patients (62). These findings thus clearly establish the osteoprotective role of Th2 lymphocytes in the pathophysiology of osteoporosis.

### Th9 Cells and Osteoporosis

Th9 cells are recently defined subset of Th cells producing IL-9 and have been associated with immune responses against intestinal worms and immunopathology of various allergic and autoimmune disorders, *viz*. systemic sclerosis, systemic lupus erythematosus (SLE) and EAE (38, 63). Th9 cells in association with Th2 cells are believed to be promoting inflammation during allergic diseases but it still lacks full validation (64). In other cases, there has been a close association between IL-9 with Th17 cells development, as IL-9 in the presence of TGF-β initiates differentiation of naive CD4^+^ T cells into Th17 cells (65). In addition, IL-9 has also been reported to amplify the development of Th17 cells during positive autocrine loop (66). In addition, serum levels of IL-9 or total Th9 cell population have been found immensely increased in case of arthritis and OA (38). Together, these studies point towards the role of Th9 cells in various bone conditions like RA but evidences are still lacking for its correlation with osteoporosis, which warrants further research in the field.

### Th17 Cells and Osteoporosis

Th17 cells are associated with protection against bacterial infection and are primarily responsible for induction of various autoimmune diseases *via* recruitment of signatory cells especially neutrophils (34, 38). The differentiation and development of Th17 cells is mainly carried by TGF-β, IL-6, IL-1β and IL-23. Primary cytokines secreted by Th17 are IL-17A, IL-17F, IL-21 and IL-22 (67). Th17 cells have also been reported to be constitutively present across mucosal surfaces including lamina propria of intestine (68). Th17 cells play an important role in various inflammatory conditions, such as osteoporosis, psoriasis, periodontal disease, RA and IBD (55, 56). Th17 cells are thus now often labeled as osteoclastogenic subsets of T lymphocytes (17, 69). Th17 cells enhance osteoclastogenesis by secreting higher levels of IL-1, IL-6, IL-17, RANKL and TNF in addition to low levels of IFN-γ (18, 70, 71). IL-17 enhances osteoclastogenesis by stimulating osteocytes and osteoblasts and potentiates osteoclastogenic activity *via* upregulation of receptor activator of NF-κB (RANK) production to produce higher levels of RANKL (57, 72). IL-17 acts as a pivotal communication point between T lymphocytes and osteocytes by modulating production of RANKL (73). Although the profile of Th17 lymphocytes in different lymphoid tissues requires further analysis, its role in the pathogenesis of osteoporosis is now well documented, since in osteoporotic patients Th17 cell population has been found to be increased many folds (34, 38). In addition, Th17 cells in the blood and peripheral tissues can serve as an important marker for osteoporosis. Altogether, these observations and studies dissect a clear and comprehensive role of Th17 cells in osteoporosis.

### Treg Cells and Osteoporosis

Regulatory T cells (CD4^+^CD25^+^Foxp3^+^ T cells) represent a special subset of Th cells that are mainly accounted for the prevention of autoimmune diseases, maintaining peripheral tolerance and limiting chronic inflammatory diseases by suppressing and regulating the effector function of Th cells (74). It has been reported that Foxp3^+^ Tregs are represented by both natural Treg (nTreg) and adaptive or induced Treg (iTreg) cell populations. nTreg cells develop in the thymus whereas iTreg cells are generated in the periphery (75, 76). nTregs represent population of CD4^+^ T lymphocytes residing in the thymus and constitute approximately 5–10% of the peripheral naive CD4^+^ T lymphocyte pool in both mice and humans. They play a significant role in the maintenance of immunological self-tolerance and modulation of immune responses (77). iTregs are found in peripheral lymphoid tissues and are derived from naive T cells (78). IL-10 and TGF-β cytokines are mainly recruited for the development and differentiation of iTregs (79). Forkhead transcription factor (Foxp3) usually expressed by Tregs has an important role in the development and functions of Tregs (77). Treg cells presume an important role in immune regulation during various inflammatory and autoimmune diseases, since Tregs regulate secretion and expression of pro-inflammatory and anti-inflammatory cytokines (18, 34, 80).

Tregs also secrete cytotoxic T-lymphocyte antigen 4, which control immune function upon binding with CD80/CD86 present on mononuclear osteoclast cells thereby leading to inhibition of inflammatory responses (34). Furthermore, inhibition of collagen-induced arthritis in mice was reported to be suppressed by Treg cells (81). Treg cells have been reported to directly inhibit osteoclastogenesis by suppressing RANKL and M-CSF production leading to increased bone volume (20, 82). Recently, the CD8 counter part of Treg cells has also been discovered as osteoclast-induced FoxP3^+^CD8^+^ Treg cells which suppress both the formation and activity of osteoclasts *via* suppression of actin ring formation leading to inhibition of osteoclastogenesis (22). Interestingly, this regulatory bi-directional mechanism does not require the presence of various pro-inflammatory cytokines. Altogether, it has now been well established beyond doubt that any dysregulation in the population or functioning of Tregs would result in enhanced bone loss reported in osteoporosis. Thus, exploring novel pathways and molecular mechanisms regulating the cross talk between Tregs and bone cells is highly desired for future clinical implications.

### T_FH_ Cells and Osteoporosis

Follicular helper T lymphocytes are usually found in the follicles of lymphoid tissue and induce production of immunoglobulins from B cells (83). T_FH_ cells express various distinctive genes such as C-X-C chemokine receptor type 5 (CXCR5), inducible T-cell costimulator (ICOS), CD40L, programmed cell death-1 (PD-1), B-cell lymphoma 6 protein and IL-21 (84). Growing evidence has shown that T_FH_ cells influence the severity of various autoimmune diseases such as RA and SLE by enhancing generation of reactive autoantibodies from B cells (85). There has been reports of increased number of circulating T_FH_ lymphocytes (*viz*. CXCR5^+^PD-1^+^CD4^+^ or CXCR5^+^ICOS^+^CD4^+^) in SLE patients which correlates with the amount of autoantibodies and SLE severity (86). Specific immunohistochemistry analysis has further confirmed the presence of T_FH_ lymphocytes (CD4^+^CXCR5^+^ICOS^+^) in the synovial tissues of RA patients (38). In addition, in RA patients, there was observed an elevation of CD19^+^ B cells and increased serum IL-21 which is positively associated with disease scores and presence of anti-citrullinated antibodies (87, 88). There has been reports of elevated levels of T_FH_ cells in both Sjogren’s syndrome patients and RA patients (88, 89). These studies provide strong evidence for potentially important roles played by T_FH_ cells in the pathogenesis and progression of autoimmune diseases through various pathways. Collectively, these studies establish the role of T_FH_ cells in RA thereby opening new avenues in the field of immunoporosis for further dissecting and delineating the role of T_FH_ lymphocytes in pathogenesis of osteoporosis.

### Natural Killer T (NKT) Cells and Osteoporosis

Natural killer T cells represent heterogeneous group of T lymphocytes which share properties of both natural killer cells and T cells. NKT cells are primarily involved in the clearance of transformed and virus-infected cells (90). NKT cells modulate initiation and development of immune responses mediated by both T and B cells *via* production of various growth factors and cytokines (16). NKT cells have been found to regulate development and function of macrophages, monocytes and myeloid dendritic cells (91, 92). A recently reported subset of NKT cells called as invariant NKT have been found to regulate the development and differentiation of osteoclasts (93). NKT cells have also been reported in the synovial tissues of RA patients where they constitute up to 20% of all the lymphocytes (94). The CD56^bright^ subset of NKT cells have been reported with upregulated expression of various chemokine receptors and adhesion molecules responsible for enhanced recruitment of NKT cells (95) thereby engaging and activating monocytes for enhanced osteoclastogenesis in synovium of RA patients (94). T cells and macrophages activated by NKT cell-derived IFN-γ also leads to increased secretion of TNF-α (96), a strong pro-osteoclastogenic cytokine. TNF-α enhances osteoclastogenesis in a RANKL-dependent manner either directly or by promoting commitment of progenitors to osteoclast lineage and differentiation or indirectly by stimulating secretion of RANKL and M-CSF by osteoblasts (97, 98). In addition, RANKL and MCSF are also produced by NKT cells and thus induce osteoclastogenesis which is further upregulated by IL-15 (99). Altogether, these reports elaborate the important role of NKT in the pathogenesis of inflammatory bone diseases. Thus, regulation of NKT cells can be an important aspect for regulating bone loss in osteoporosis.

### γδ-T Cells and Osteoporosis

T cells present in circulation mainly express αβ-T cell receptor (TCR) chains along with a small subset of T cells which uniquely expresses TCRs, containing a gamma (γ) chain and a delta (δ) chain named as γδ-T cells. About 1–10% of T lymphocytes in periphery of human circulation comprise of γδ-T cells, however, skin and other tissues have more abundant population of γδ-T cells (99). γδ-T cells are more innate-like, unlike their counterpart αβ-T cell which are adaptive. Also, the TCR specificity of γδ-T cells is uniquely directed toward non-peptide antigens. γδ-T cells are now increasingly being linked with autoimmunity, allergy, hematological tumors (100) and infectious diseases (101). γδ-T cells have recently been reported to produce factors which are important in the healing of various skeletal fractures (102). It has been observed that anti-CD3/CD28-stimulated γδ T cells lead to inhibition of human osteoclast formation and simultaneous resorptive activity *in vitro*. In addition, stimulated γδ-T cells leads to increased production of IFN-γ and inhibits expression of IL-17 production (103). γδ-T cells are quite unique and heterogeneous population of T lymphocytes and are easily lost in patients undergoing amino-bisphosphonate treatment for various bone-related pathologies (104). γδ-T cells have been associated with peculiar property of immunomodulation and thus are a promising candidate for treatment of various inflammatory conditions including bone pathologies.

### CD8^+^ T Cells and Osteoporosis

CD8^+^ T cells are yet another established subset of T lymphocytes which play a key role in cell-mediated responses in the immune system. CD8^+^ T cells are also referred as cytotoxic T lymphocytes which aid in protection of host from foreign organisms through both lytic and non-lytic means. CD8^+^ T cells are responsible for regulating the immune responses and simultaneously eliminating transformed tumor cells (104, 105). Another recently defined regulatory subset of CD8^+^ T cells called as Foxp3^+^CD8^+^ Tregs has been found to suppress osteoclast formation and activity by secreting various anti-osteoclastogenic cytokines (22). Foxp3^+^CD8^+^ Tregs cells not only regulate the survival of osteoclasts but also affect the maturation of osteoclasts by suppressing their actin ring formation. The recently discovered unique property of osteoclast inducing the generation of FoxP3^+^CD8^+^Treg cells and the ability of FoxP3^+^CD8^+^Treg cells to subsequently regulate osteoclast function establishes a bi-directional regulatory loop between these two cells types in the BM (22). CD8^+^ T cells have also been reported to play an important role in the pathogenesis of OA (38). These reports thus clearly establish the important role of CD8^+^ T cells in bone health, but still evidences for their role in osteoporosis is not well established and needs further research.

## Conclusion and Future Perspectives

**Figure 3:**
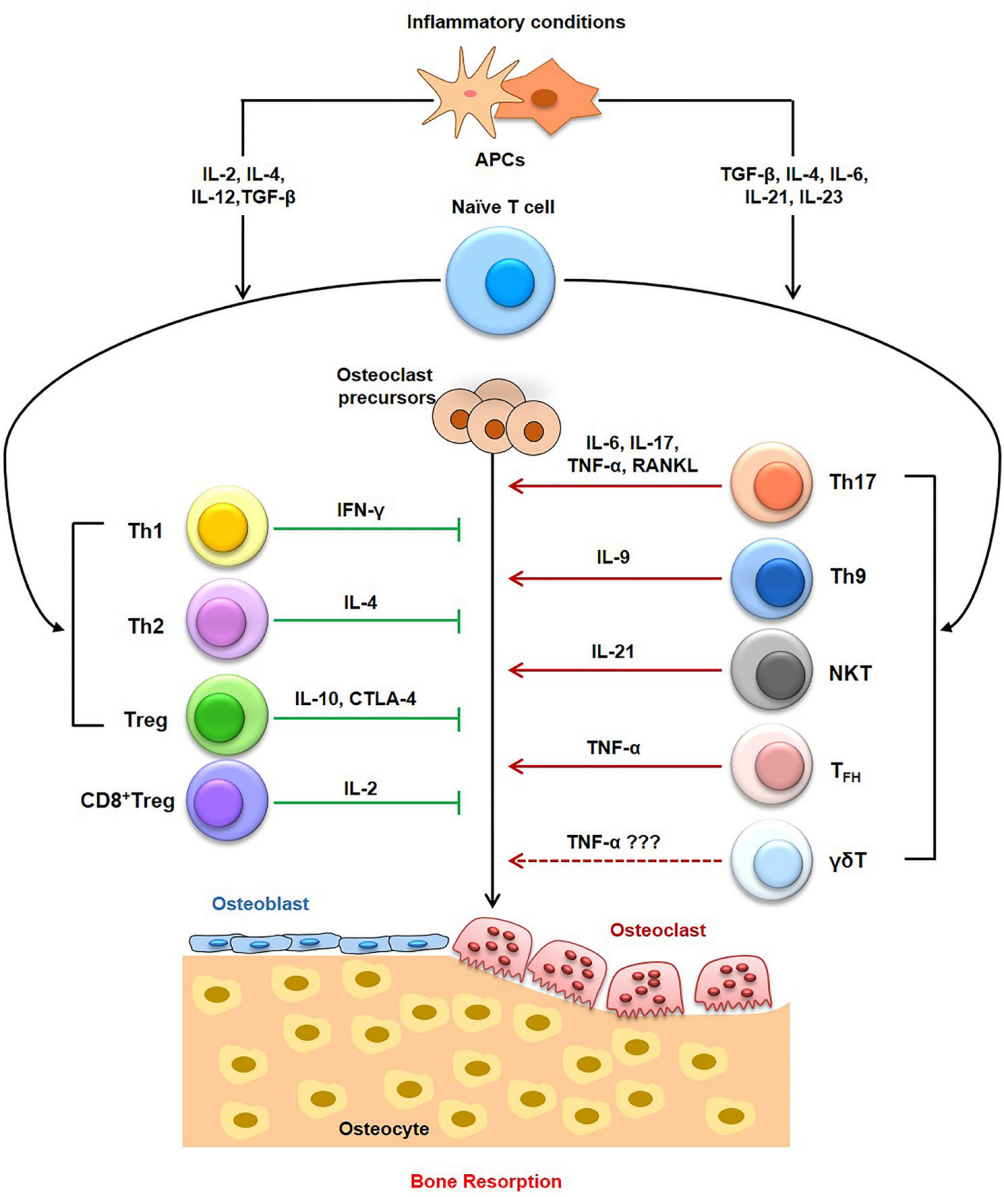
Role of T cells in Immunoporosis. T cells, including the T helper (Th) cells, cytotoxic T cells have pivotal role in the induction of osteoporosis-associated bone loss. Within T helper (Th) cells, Th9 cells, Th17 cells, NK T cells, and follicular helper T (T_FH_) cells have been reported to enhance bone loss in osteoporosis. On the other hand, Th1, Th2, Tregs, and CD8T cells have been associated with osteoprotective properties, thereby inhibiting osteoporosis. The role of γδ T cells need more studies to relate them with osteoporosis.

Inflammatory bone conditions including RA, OA and osteoporosis arise due to dysregulation of the homeostatic nexus between bone and immune system thereby leading to enhanced bone loss. Various strategies are now being developed for inhibiting inflammation induced bone loss. One direct approach of inhibiting osteoclastogenesis could be interfering with inflammatory pathways thereby providing an alternate method for managing inflammatory bone loss/damage. The emergence of “immunoporosis” as an independent field of research would thus give a novel and unique insight into the underlying immune-skeletal interaction between both immune cells (T cells) and bone cells (Figure 3). Since the role of immune system, in general, has been implicated for elucidating the pathogenesis of numerous inflammatory diseases, however, the immunoporosis aspect, i.e., the role of specific subsets of T lymphocytes in osteoporosis is still unclear. Thus, it will be important to see the course of various subsets of T cells in the development and progression of osteoporosis. Both CD4^+^ (Th1, Th2, Th9, Th17, Treg, NKT, γδ T cell subsets) and CD8^+^ T cells play an important role in regulating bone health. Th17 cells are one of the major inducers of bone loss *via* expression of higher levels of RANKL and TNF-α (17, 106, 107). On the other hand, Tregs are major inhibitors of bone loss (108, 109) through production of IL-4, IL-10 and TGF-β1 cytokines (108, 109). Tregs also inhibit the effector functioning of Th17 cells in inflammation-induced bone loss (109, 110). Tregs also lead to suppression of bone loss by inhibiting differentiation of monocytes into osteoclasts under both *in vitro* and *in vivo* conditions (20, 75, 111) (Table 1).

**Table 1:** Role of various T cell cytokines in osteoclastogenesis.

Cytokine	Source	Modulation of immunity	Osteoclastogenic function	Reference
Interleukin (IL)-4	Th2	Humoral immunity	Inhibits osteoclastogenesis	(115)

IL-6	Macrophage, dendritic cells (DCs)	Pro-inflammation, Th17 induction	Activation of osteoclastogenesis	(116)

IL-10	Regulatory T (Treg)	Anti-inflammatory	Suppress bone resorption	(117)

IL-15	NK cells	Pro-inflammatory cytokine	Enhances RANK ligand (RANKL) and macrophage colony-stimulating factor expression	(99)

IL-17	T cells	Pro-inflammatory cytokine	RANKL expression and vigorous pro-inflammatory potency	(118)

IL-18	Macrophage	Th1 differentiation, interferon (IFN)-γ Induction	Inhibits TNF-α mediated osteoclast	(119)

IL-23	Macrophage and DCs	Th17 induction	Indirect osteoclast activation	(17)

IL-27	Macrophage and DCs	Th1and Treg Th17 induction	Inhibits osteoclast formation, blocking receptor activator of NF-κB (RANK)-dependent osteoclastogenesis	(120)

MCSF	Th1	Pro-inflammatory	Inhibits osteoclastogenesis	(121)

IFN-γ	Th1, NK cells	Cellular immunity	Inhibits osteoclastogenesis	(116)

Osteoprotegerin	Osteoclasts	Decoy receptor for RANKL	Inhibits osteoclastogenesis	(122)

RANKL	Th17 cells	Osteoclast differentiation DCs maturation	Osteoclast activation *via* RANK	(123)

RANK	Osteoclasts, DCs	DCs activation	Osteoclast differentiation and activation	(121)

TNF-α	Th17, macrophage DCs	Pro-inflammatory cytokine	Indirect osteoclastic activation through RANKL	(123)

Transforming growth factor beta	Multiple cell lines	Blocks activation of lymphocytes and monocytes derived phagocytosis	Indirect osteoclast activation, Inhibits osteoblast differentiation	(124)

Since osteoporosis affects more than 50% of female population and one-fourth of males above age 50 (3, 20), osteoporotic patients are very higher in number than OA and RA (3). The current therapies employed for the treatment of osteoporosis, *viz*. bisphosphonates, strontium ranelate, selective estrogen receptor modulators, PTH (teriparatide), etc. provide remission of inflammation with little relief to the patients (112). Therefore, the need for new and effective future therapeutics are need of the hour for long-term relief from various bone loss mediated pathologies. This could be made possible only upon having a clear and better understanding of immune-skeletal biology which can be clearly defined and delineated under the novel field of immunoporosis. Thus, advanced exploration and research under the aegis of immunoporosis would lead to novel opportunities and avenues for development of enhanced therapies. Recently, the inclusion of probiotics (*via* modulation of host immune system) as a supplementary therapy for bone loss represents one such class (20, 113, 114). Thus, a better understanding of the nexus between both the systems should be at heart of future research in the area. The establishment of “immunoporosis” as an independent field of modern biology catering to the recent developments in the field will thus provide new paradigms for development of focused novel therapeutic strategies for managing osteoporosis.

## Author Contributions

RS suggested the focus and outline of the review along with writing the review. HD participated in the writing of the review along with creating figure illustrations. PM provided valuable inputs for manuscript preparation.

## Conflict of Interest Statement

The authors declare that the research was conducted in the absence of any commercial or financial relationships that could be construed as a potential conflict of interest.

## Abbreviations

IFN, interferon; MCSF, macrophage colony-stimulating factor; NF-κB, nuclear factor κB; BM, bone marrow; T_FH_, follicular helper T; NKT cells, natural killer T cells; OPG, osteoprotegerin; TRAF, TNF receptor-associated factor; TRAF-6, TNF receptor-associated factor 6; RANK, receptor activator of NF-κB; RANKL, RANK ligand; MCP-1, monocyte chemoattractant protein-1; TGF-β, transforming growth factor beta; BMD, bone mineral density; TCR, T cell receptor; Th, T-helper; DC, dendritic cell; RA, rheumatoid arthritis; OA, Osteoarthritis.
